# Unfolding the Semen Microbiota: Implications for Male Infertility

**DOI:** 10.3390/biomedicines14071557

**Published:** 2026-07-11

**Authors:** Michela Buttà, Arianna Sucato, Giuseppina Capra

**Affiliations:** 1 Department of Health Promotion, Mother and Child Care, Internal Medicine, and Medical Specialties G. D’Alessandro (PROMISE), University of Palermo, 90127 Palermo, Italy; michela.butta@unipa.it (M.B.); arianna.sucato@unipa.it (A.S.); 2 Microbiology and Virology Unit, Polyclinic Hospital “P. Giaccone”, 90127 Palermo, Italy

**Keywords:** male infertility, microbiota, NGS, HPV, virome, seminal microbiota, sperm

## Abstract

In recent years, the growing interest in microbiota, supported by increasing evidence from next-generation sequencing (NGS)-based studies, has led to the hypothesis that multifactorial conditions such as male infertility may have their bases in its imbalance. Despite rapid advances in the field over the past decade, findings remain fragmented and sometimes inconsistent. Furthermore, analyses frequently exclude non-bacterial components, such as viruses (e.g., human papillomavirus and herpes simplex virus), protozoa (e.g., *Trichomonas vaginalis*) and fungi (e.g., *Candida* spp.), with a few studies focusing on the influence of individual components. This review provides an integrative and critical synthesis of current knowledge on the composition and functional relevance of the male reproductive tract microbiota, including both bacterial and non-bacterial components. Particular attention is given to the methodological strengths and limitations of NGS approaches, the main bacterial taxa identified in seminal samples, and the emerging roles of viruses, protozoa, and fungi. The review also explores proposed mechanisms linking microbial dysbiosis to impaired spermatozoa function, the potential anatomical origins of seminal microorganisms, and evidence for microbiota exchange between sexual partners. By integrating recent data and addressing underexplored components beyond bacteria, this work provides a coherent framework to support future research on the role of the seminal microbiome in male infertility.

## 1. Introduction

In recent years, the scientific literature has shown increased interest in analyzing the human microbiota, the complex microbial community inhabiting the different parts of our body.

This field has grown mainly due to the development of next-generation sequencing (NGS) techniques, which enable the simultaneous analysis of hundreds of millions of sequences. This approach has been applied to a wide range of biological matrices from various anatomical regions, with a simple yet ambitious aim: to identify a possible cause of multifactorial diseases in an altered balance of the microorganisms populating our bodies.

Despite the relatively easy production of data, interpretation has often been tricky. The first issue concerns the causal relationship between the occurrence of the illness and the disturbance in the microbiota equilibrium. In this claim of the egg first or the hen, data have continued to increase, and right now the scientific landscape shows countless studies supporting the theory that microbial dysbiosis is strictly linked to specific pathological states.

Bacteriospermia, as identified using the traditional culture-dependent approach, was originally associated with infections of the urogenital tract. It was correlated with acute and chronic prostatitis, as well as with urethritis, orchitis, and epididymitis [1]. However, the outdated idea that the presence of bacteria in semen always indicates an infectious state is gradually being dispelled. In fact, advanced sequencing techniques have revealed that the upper genital tract, and therefore sperm, is not sterile. The ‘Human Microbiome Project’, which aims to characterize the human microbiome, has shown that the urogenital tract harbors 9% of the total human microbiota [2].

However, compared to other anatomical sites, such as the vagina or gastrointestinal tract, the microbiota of the male genital tract is an understudied subject, with the first study specifically addressing semen microbiota dating back to 2013 [3]. Studying the seminal microbiota is relevant to a variety of issues, including prostate diseases (e.g., prostatitis, benign prostatic hyperplasia, and prostate cancer) and infertility.

The prevalence of male infertility has increased substantially, with a marked rise observed between 1990 and 2019 [4]. It is estimated that male factors are the main cause of infertility in about 30% of couples, and in at least half of these cases, they are a contributing factor [5]. Nonetheless, the etiological or pathophysiological underlying cause is often overlooked. From this, arises the high rates of so-called “idiopathic” infertility cases.

When searching for this condition, the coventional clinical approach involves the examination of semen characteristics described in the sixth edition of the WHO laboratory manual for the examination and processing of human semen, dating back to 2021 [6]. As part of the basic examination, the operator assesses the macroscopic characteristics of semen, including appearance, volume, viscosity, and pH of the semen, and subsequently performs a microscopic analysis to evaluate spermatozoa parameters such as count, concentration, vitality, and motility, categorized as progressive, non-progressive, or immotile, and morphology, with abnormalities described in the head, midpiece, or tail. However, in some cases, normozoospermic individuals, i.e., without abnormalities of the semen, remain unable to conceive. These cases of idiopathic infertility are investigated by the evaluation of other parameters, including the analysis of spermatozoa DNA fragmentation, assessment of interleukins indicative of inflammation, presence of anti-spermatozoa antibodies (ASAB), reactive oxygen species (ROS) or leukocyte cells. It may not be surprising that a ‘dysbiosis’, i.e., a variation in the composition or abundance of the entire microbial community or specific microorganisms, could cause infertility, with or without alterations to the spermogram [7].

Despite the rapid proliferation of reviews on the seminal microbiome, most of the existing literature remains focused almost exclusively on bacterial community composition. A significant knowledge gap exists regarding non-bacterial components: viruses, fungi, and protozoa are frequently excluded or treated as isolated clinical pathogens rather than integral parts of a complex microbial ecosystem.

In light of this, the aim of this narrative review is to provide a comprehensive and integrative synthesis of seminal microbiota research, focusing not only on bacterial communities but also on the often-overlooked contribution of viruses, fungi, and protozoa. The bacteria-focused section will be centered on NGS-based studies, which, although still emerging, are rapidly expanding. In contrast, the discussion of other microorganisms will focus on their potential role as pathogens, given the total lack of fungal and viral assessments in most NGS-based infertility studies. The review will also describe potential mechanisms underlying impaired semen functionality and will include a dedicated section addressing the methodological limitations of microbiota-focused studies.

The literature search was conducted in PubMed to identify relevant studies on seminal microbiota and male reproductive health. The search was performed using combinations of the following keywords: “seminal microbiota”, “semen microbiota”, “male infertility”, “seminal virome”, “seminal mycobiome”, and “protozoa” combined with “male infertility”. The search was restricted to articles published in English, applying a predefined time limit going from 2015 to 2025 Additional relevant studies were identified through manual screening of reference lists of selected articles to ensure comprehensive coverage of the topic. Given the narrative nature of this review, a formal PRISMA protocol was not applied; however, the search strategy was designed to ensure transparency and reproducibility of the included literature.

## 2. Methodological Approaches and Limitations in Seminal Microbiota Studies

The study of seminal microbiota began with research based on a classical cultivation-dependent approach, which provided a narrow picture of the bacterial population, excluding the unculturable portion. The emergence of NGS techniques has allowed us to expand the picture, albeit introducing new technical and interpretive challenges.

The most common approach to identifying bacteria involves the sequencing of 16S rRNA gene hypervariable regions amplicons, including in the analysis culturable and unculturable species, from the least represented to the more abundant. This approach can limit the results by excluding certain taxa from amplification, as the universal primers used may not be equally effective across all bacterial groups. Conversely, the high variability of the targeted regions may still be insufficient to reliably distinguish closely related species.

An alternative approach is shotgun metagenomic sequencing rather than amplicon sequencing. Shotgun metagenomics samples DNA fragments from across the microbial community, not just a targeted marker gene, and therefore can provide broader taxonomic and functional information.

However, in both workflows, taxonomic assignment commonly relies on comparison with reference databases. Therefore, sequences from previously uncharacterized microorganisms may be detected but not confidently assigned to a known taxon. In such cases, they may remain unclassified or be assigned only to a higher taxonomic rank [8].

In addition to these intrinsic technological constraints, a critical and often underappreciated limitation in seminal microbiota studies is contamination introduced during sample collection, handling, and laboratory processing. Semen is a low-biomass biological matrix, which makes it particularly susceptible to the influence of exogenous DNA. Contamination may occur at multiple stages, including during sample collection (e.g., skin-associated bacteria introduced via genital contact, inadequate genital cleaning prior to masturbation, contamination from collection containers, or inadvertent exposure to environmental microbiota), as well as during clinical handling and transport [9]. Moreover, laboratory-derived contamination represents a well-recognized issue in microbiome research, including DNA extraction kits, PCR reagents, laboratory surfaces, and sequencing batch effects, all of which may introduce background microbial signatures that can be misinterpreted as true biological signals. In seminal samples, even minimal contamination can substantially distort microbial profiles, leading to overestimation of diversity or false detection of taxa that may in fact represent reagent or environmental contaminants [10].

These biased interpretations could be minimized by taking common measures to guarantee the cleanest possible sample collection and processing: the experiment should be conducted in a UV- and sodium hypochlorite solution-treated environment, using ethylene oxide-treated consumables and wearing clean protective equipment. The different steps (i.e., extraction and library preparation) should be performed in separate rooms, and to randomize samples and treatment, it is recommended to avoid batch effects and daily variations in contaminants [11].

Moreover, every phase of the process should include negative controls: sampling and DNA extraction blank controls, and a no-template control during PCR.

This approach, albeit rarely included in microbiome studies, allows the recognition of contaminants, which can be filtered during the bioinformatic analysis [11].

In addition to the aforementioned issues, there is a substantial methodological heterogeneity among studies, which further limits comparability. The sequencing platforms used vary greatly (e.g., Roche 454, Illumina MiSeq, and Ion Torrent), as do the hypervariable regions analyzed (e.g., V1–V2, V3–V4, and V1–V9), and the bioinformatic pipelines employed (QIIME 2, DADA2, mothur, HUMAnN).

This heterogeneity could also regard the cohort’s characteristics in terms of geographical origin, diet, sexual habits and exposure to pollutants, which are known factors that influence microbiota composition [12]. In particular, studies are often inconsistent in reporting sexual behavior variables: condom use, sexual practices (e.g., oral or anal sex), sexually transmitted infection history, and partner-related characteristics are often not systematically collected or reported. In line with the SAGER guidelines, future research should incorporate standardized reporting of these variables to improve methodological rigor and reduce potential bias [13].

Equally limited and inconsistent is the reporting of clinical conditions affecting male reproductive health, such as urogenital disorders (e.g., varicocele), as well as infections and inflammatory conditions, that may also influence semen parameters and potentially confound microbiota analyses [14].

Another challenge in defining the exact composition of the seminal microbiota is distinguishing between stable community members and transient elements, which can only be reliably identified through well-designed longitudinal studies that remain limited at present.

It is important to note that bacteria are not the only microorganisms that make up the microbiota. Viruses, fungi, and protozoa also play a role. However, studies focusing on these subjects are scarce, as a shared but variable region equivalent to the bacterial 16S region does not exist, except for fungi and their ITS (Internal Transcribed Spacer) region.

Consequently, studies on the seminal virome are virtually non-existent, with the only available papers focusing on the effects of a single virus on male infertility. In a similar fashion, fungi and protozoa are frequently marginalized in the discourse, with analysis once more concentrated on the impact of a solitary element on reproductive health.

## 3. Mechanisms Linking Seminal Microbiota to Male Infertility

The ways by which dysbiosis could impair spermatozoa parameters and/or reproductive potential are not fully understood. One of the most widely described mechanisms involves the activation of an inflammatory response coupled with cytokine secretion and leukocyte infiltration. This is associated with increased ROS production, resulting in oxidative stress (OS), which may potentially affect spermatozoa DNA integrity through strand breaks, as well as chromatin packaging via alterations in protamination and histone-to-protamine replacement. It may also compromise the integrity of mitochondrial and plasma spermatozoa membranes, which, being particularly rich in unsaturated fatty acids, are more sensitive to oxidative damage [15,16].

This inflammatory milieu may also affect the patency of the ejaculatory ducts and promote fibrotic tissue remodeling, thereby altering gland secretion [17]. Additionally, inflammation-induced disruption to the blood–testicular barrier can stimulate the production of ASABs, thereby impairing spermatozoa function [18].

It is interesting to note that leukocytospermia (i.e., the presence of leukocytes in semen), a strong indicator of an inflammatory response, has been identified in only 15% of subfertile men with no symptoms of genitourinary infection. Furthermore, it has not been significanly associated with bacteriospermia or seminal dysbiosis [19,20].

Similar inflammatory mechanisms may also be triggered by viral infections, both genital and systemic. Moreover, viral replication itself may disrupt the blood–testis barrier and damage cells in the urogenital tract, negatively affecting their integrity and function, as well as their DNA stability and epigenetic regulation [21]. As for systemic infection, febrile episodes inducing even a slight increase in testicular temperature can harm spermatogenesis [22]. Notably, inflammation of the male genital tract is often asymptomatic, which may result in a prolonged subclinical state potentially detrimental to fertility [23].

Beyond inflammation, bacteria may also impair spermatozoa function through direct mechanisms independent of the inflammatory response, including adhesion to spermatozoa or the production of soluble factors such as toxins, enzymes, and ROS [24]. For instance, hemolysins can damage cell membranes, and a spermatozoa immobilization factor can impair spermatozoa mobility and viability [23]. Furthermore, bacteria can induce a decrease in mitochondrial membrane potential, leading to protamine deficiency, disruption of Protamine 1/Protamine 2 (P1/P2) ratio, and spermatozoa cell apoptosis [12,25].

**Figure 1:**
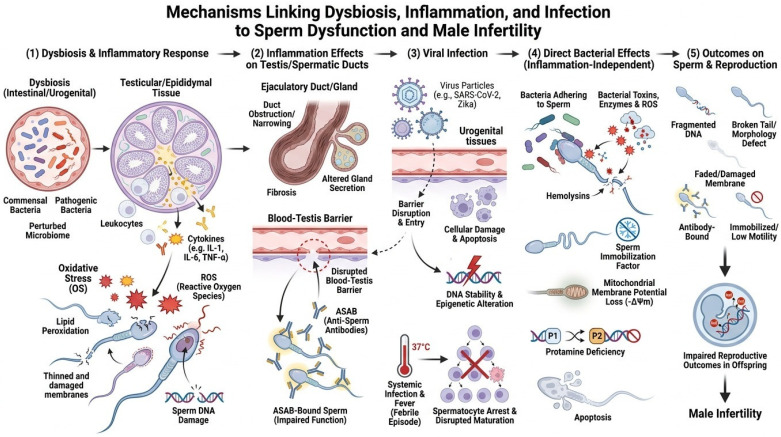
Proposed mechanisms which may explain the link between dysbiosis, inflammation and infection to spermatozoa dysfunction and male infertility.

Regardless of the mechanisms involved, spermatozoa impairment could involve parameters beyond those classically evaluated during semen examination, such as epigenetic changes, potentially affecting gene expression in offspring and negatively influencing reproductive outcomes (Figure 1).

It should be noted, however, that while several studies support associations between seminal microbiota alterations and inflammatory or oxidative stress markers, many of the downstream mechanisms linking these processes to spermatozoa DNA damage remain hypothetical and are supported by different types of experimental evidence. Inflammation and oxidative stress are supported mainly by human observational studies [25,26,27], while direct bacterial injury to sperm, including adhesion/agglutination, immobilization factors, apoptosis, and reduced mitochondrial membrane potential, comes mainly from in vitro work, with some human corroboration through associations with protamine deficiency and DNA fragmentation in bacteriospermia [25]. Finally, blood–testis barrier disruption, fibrosis/ductal remodeling, endocrine-epigenetic effects, and reproductive outcomes are supported mostly by animal, ex vivo, or indirect human data; pregnancy and live-birth evidence remains sparse [25,26].

Despite the growing number of studies reporting associations between seminal dysbiosis and impaired reproductive outcomes, the direction of this relationship remains uncertain. Most available evidence derives from cross-sectional studies, which do not allow causal inference. Therefore, microbial dysbiosis may represent a contributing factor to male infertility through inflammation, oxidative stress, immune activation, and direct spermatozoa damage, but it may also arise as a consequence of alterations in the reproductive tract associated with infertility. A bidirectional interaction is also plausible, whereby infertility-related changes promote microbial imbalance, which in turn exacerbates reproductive dysfunction. Clarifying these relationships will require longitudinal studies and mechanistic investigations capable of tracking microbiota dynamics over time and establishing temporal associations with changes in reproductive health.

## 4. Key Bacterial Agents: A Profile of the Most Common Infertility Associated Bacteria

In an understudied field such as the seminal microbiota, the most frequently addressed subject is its composition in bacterial species.

From the earliest culture-based studies to the most recent NGS investigations, a variety of aerobic, anaerobic and microaerophilic bacteria have been identified. However, differently to the female counterpart, for which extensively conducted studies describe well-defined Community State Types (CSTs) inhabiting the vagina niche, the same cannot be said for seminal bacterial microbiota.

Only some NGS-based studies have been able to define an association between bacterial taxa-enrichment and alteration of the seminal characteristics, and with not completely coherent findings. However, it seems that a core set of phyla consistently emerges: *Firmicutes* is the dominant phylum, followed by *Proteobacteria*, *Bacteroidota*, and *Actinobacteriota*. At the genus level, the most frequently reported genera are *Lactobacillus*, *Prevotella*, *Streptococcus*, *Staphylococcus*, and *Corynebacterium*, although their relative abundances vary across populations and clinical conditions [3,20,28,29,30,31,32,33,34,35,36,37,38] (Supplementary Table S1).

Almost all the studies on the subject drew the conclusion that what varies significantly is the differential abundance of specific taxa, i.e., which specific bacteria are more or less abundant under a given condition (e.g., fertile vs. infertile males or normal vs. abnormal seminal parameters), rather than alpha diversity (i.e., species diversity within a sample) or beta diversity (i.e., the difference in composition between samples).

These two parameters, whose measurement represent the first step in every microbiota-focused research, rarely emerged as significantly altered. This happened in the studies by Baud et al. [31], Garcia-Segura et al. [36], Cao et al. [39], and Mowla et al. [40], who reported no significant differences in both parameters between fertile and infertile men, regardless of the specific semen abnormality.

Some exceptions are the studies by Yao et al. [35], which observed significantly lower alpha diversity in men with primary idiopathic infertility compared to fertile controls, or Yang et al. [32], who demonstrated significant compositional differences between asthenozoospermic or oligoasthenozoospermic men and healthy controls, although they failed to identify alpha diversity differences. Similarly, Chen et al. [29] reported reduced diversity in men with non-obstructive azoospermia (NOA), although this was not confirmed by Campbell et al. [41] in a larger NOA cohort. In the latter study, authors found no global differences in beta diversity between the NOA group and the control group. However, they did observe altered co-occurrence networks, which suggests that it is community interactions rather than overall composition that are disrupted.

Notably, despite not being associated with fertility status, beta diversity was able to distinguish between microbiota clusters (e.g., *Streptococcus*-, *Prevotella*- and *Lactobacillus*-dominated profiles) in both Baud et al. [31] and Mowla et al. [40], with *Prevotella*-enriched clusters also apparently exhibiting higher alpha diversity and bacterial load. Similar bacterial communities have previously been described by Hou et al. [3] and Weng et al. [28], two of the first NGS-based studies on the subject, which in their cohort of infertile men were able to distinguish three different communities: *Prevotella*-predominant, *Pseudomonas*-predominant, and *Lactobacillus*-predominant. The same authors agreed in identifying a correlation between *Lactobacillus* species and better seminal parameters, while the abundance of *Prevotella* seemed to have the opposite effect.

The detection of *Prevotella* genus has been repeatedly associated with poor semen quality, infertility and impaired spermatozoa in numerous studies [7,31,34,35,40]. Although not yet diagnostically validated, it is one of the taxonomic groups with the highest level of cross-validation across independent studies, different geographical populations and various methodologies. Specifically, Baud et al. [31] and Yang et al. [32] reported an increase in *Prevotella* in spermatozoa with impaired motility, while Cao et al. [39] described a negative correlation between the genus and spermatozoa concentration and total spermatozoa count. Furthermore, morphological alterations have been observed in *Prevotella*-enriched samples [28,31].

More generally, infertility status was associated with a high *Prevotella* load in the studies by Okwelogu et al. [34] and Gachet et al. [7], respectively in patients with negative in vitro fertilization (IVF) outcome and in samples associated with negative bacterial cultures. In this context, *Prevotella bivia* and *Prevotella timonensis* were among the five most abundant species in infertile men analyzed by Yao et al. [35].

The negative effects of *Prevotella* are often attributed to its competition with *Lactobacillus* and to its LPS, which could trigger an inflammatory response and consequent DNA damage (Table 1).

Only a few studies, like those by Monteiro et al. [30], Garcia-Segura et al. [36] and Campbell et al. [41] despite identifying the *Prevotella* genus did not find any association with a specific spermatozoa alteration.

As for the *Lactobacillus* genus, most of the available literature describes it as being associated with normal semen parameters and normal reproductive function. In several independent cohorts, a *Lactobacillus*-enriched microbiota profile has been linked to normozoospermia, better spermatozoa motility, concentration, normal morphology and reduced spermatozoa DNA fragmentation [28,31,36,42]. On the contrary, lower *Lactobacillus* spp. concentration has been described as negative correlated with leukocyte concentration and spermatids percentage [42] (Table 1).

The mechanisms underlying the apparently beneficial association of lactobacilli with reproductive health remain incompletely understood. Several hypotheses have been proposed, primarily based on studies of the gut and vaginal microbiome. However, these have not yet been demonstrated experimentally for semen.

Lactobacilli are known producers of bacteriocins, small antimicrobial peptides that suppress the proliferation of pathogenic bacteria, whose colonization may be further limited through competitive exclusion and niche occupation [43,44]. Through these mechanisms, lactobacilli may help shape a microbial environment less permissive to the overgrowth of potentially pathogenic bacteria. In turn, a lower abundance of pathogenic taxa has been associated in several studies with reduced local inflammatory activity and decreased leukocyte infiltration, which may indirectly limit ROS production and oxidative stress [45].

In addition, evidence from mucosal microbiome research suggests that lactobacilli may modulate host immune responses, potentially influencing the balance between pro- and anti-inflammatory mediators. Again, most of these immunomodulatory effects have been described in gut and vaginal models, and their direct relevance to the seminal environment remains to be fully established [129].

By limiting inflammation and ROS production, these mechanisms may protect spermatozoa from DNA damage and plasma membrane lipid peroxidation, thereby preserving spermatozoa structural integrity and function [40,46,47].

However, all of the described mechanisms are largely speculative, and the beneficial role of *Lactobacillus* spp. is called into question by a few studies, among which the paper by Yang et al. [32] distinctively stands out as the most significant outlier. The authors, indeed, found increased *Lactobacillus* abundance in asthenozoospermic and oligoasthenozoospermic patients, with the genus achieving a high diagnostic accuracy for these conditions. This apparent contradiction may reflect methodological differences, such as the use of distinct 16S rRNA hypervariable regions, geographical variations in diet and lifestyle, or, most importantly, the inability of genus-level analysis to capture species-specific roles.

Indeed, as has been observed in vaginal microbiota, where *Lactobacillus crispatus*, *L. gasseri* and *L. jensenii* are generally considered to be markers of an eubiotic environment, while *L. iners* displays a more ambiguous role and is often associated with transitions towards dysbiosis, it has been hypothesized that species-specific effects may also exist within the seminal microbiota.

*Lactobacillus iners* has been associated with abnormal motility and reduced spermatozoa concentration [46], whereas *Lactobacillus crispatus* and *Lactobacillus jenseni* were significantly associated with normal spermatozoa morphology [130]. These different correlations can be explained by *L. iners* producing L-lactic acid, which can induce a pro-inflammatory microenvironment, and by *L. jenseni* secreting H_2_O_2_, effective in inhibiting pathogen proliferation [34,40].

Therefore, analyses conducted only at the genus level may obscure important biological differences between *Lactobacillus* species and contribute to the inconsistent associations reported across studies (Table 1).

Taken together, evidence supports a predominantly protective role for *Lactobacillus* in male fertility but highlights that future studies should focus on performing a species-level analysis to clarify the contributions of individual species and overcome discordant findings.

The same can be said for the *Pseudomonas* genus: frequently detected both in semen characterized by normal and abnormal seminal parameters [7,28,29,30,33], the species-level analysis by Osadchiy et al. [46] let emerge how *P. stutzeri* and *P. fluorescens* are enriched in samples with abnormal spermatozoa count, while *P. putida* is more abundant in normal samples (Table 1).

A specific role has not been defined even for the *Staphylococcus* genus, with several studies not finding any association with any condition [7,32,34,35,46]. Baud et al. [31] reported that *Staphylococcus* was significantly enriched in men with normal spermatozoa parameters, suggesting a potential protective or commensal role. In contrast, Cao et al. [39] found that *Staphylococcus* abundance was significantly increased in the semen of men with asthenozoospermia and seminal hyperviscosity, supporting a detrimental effect. The same role was suggested by Bukharin et al. [33], who reported that *Staphylococcus* isolates from infertile men exhibited higher bacterial load and enhanced biofilm-forming capacity compared to isolates from fertile controls, pointing to a potential pathogenic mechanism. Again, the lack of species-level resolution in most studies prevents discrimination between commensal species such as *Staphylococcus epidermidis*, and potentially pathogenic species such as *Staphylococcus aureus*. The fact that *Staphylococcus* is a skin commensal raises the possibility that its presence in semen may reflect contamination during sample collection rather than genuine colonization of the reproductive tract (Table 1).

The available literature on the genus *Anaerococcus* suggests a potentially detrimental association with semen quality and male reproductive parameters. Since one of the earliest 16S rRNA-based studies on seminal microbiota [3], *Anaerococcus* has been recognized as negatively associated with overall semen quality, including spermatozoa concentration, motility, and volume. More recently, Yang et al. [32] found that *Anaerococcus* was significantly enriched in the semen of men with asthenozoospermia compared to fertile controls, which further supports the idea that it plays a detrimental role. These findings are compatible with what is known about the vaginal environment, in which *Anaeroccoccus* was found to be associated with bacterial vaginosis [48]. However, nor Baud et al. [31], Monteiro et al. [30], or Gachet et al. [7] studies, identified *Anaerococcus* as a differentially abundant taxon between fertile and infertile men or between normal and abnormal seminal parameters. The different geographical regions (Chinese vs. European and American) as well as the different hypervariable regions analyzed (V1–V2 vs. V3–V4, V1–V4) can explain these inconsistencies (Table 1).

The *Enterococcus* genus has emerged as a dominant taxon in several studies, in which it is frequently described as having a negative impact on fertility. Cases of idiopathic infertility, azoospermia, high spermatozoa DNA fragmentation and high biofilm production, were highlighted as significantly associated with *Enterococcus* presence [30,33,34,36,39]. In other studies, it is the dominant species in all samples, regardless of fertility status [30]. This evidence suggests that *Enterococcus* may be an opportunistic pathogen that contributes to damage to the semen in cases of dysbiosis or when particularly virulent strains are present. Its ubiquity makes it difficult to establish a pathogenicity threshold, and the lack of species-level analysis (i.e., distinguishing *E. faecalis* from *E. faecium*) limits the conclusions that can be drawn (Table 1).

One aspect to point out is that studies rarely address seminal parameters other than the classical ones. This means that evaluations which could reveal crucial information, such as sperm DNA fragmentation, are excluded.

An exception in this respect is the study by Gracia Segura et al. [36], who conducted a study analyzing DNA fragmentation index (DFI) and oxidative stress parameters, letting emerge significant associations. The authors analyzed 42 semen samples from patients with idiopathic infertility and 14 samples from donors. They performed full-length 16S rRNA gene sequencing using the Illumina MiSeq platform and evaluated spermatozoa quality parameters, chromatin integrity and oxidation–reduction potential. *Moraxella*, *Brevundimonas* and *Flavobacterium* were negatively correlated with DNA fragmentation, with *Brevundimonas* also being associated with lower oxidative reduction potential, while *Actinomycetaceae*, *Ralstonia* and *Paenibacillus* were found to be associated with poor protamination and increased dsDNA fragmentation.

Another crucial proof regarding the effects of microbiota on DFI comes from the recent paper by He et al. [131]. The authors, in fact, carried out cross-incubation experiments consisting of the incubation of high DFI seminal plasma with donors’ seminal samples, which faced an increase in DFI as well. Dividing the cohort into two different groups of high DFI and low DFI samples, the authors identified a specific rise in *Acinetobacter* species, producers of DNA-damaging metabolites (i.e., mitomycins and quinoxalines) in the former group. In the same group, also lactobacilli were highly represented, with *Lactobacillus iners* identified as the most abundant species.

On a separate note, bacteria which fall in the category of sexually transmitted diseases (STDs) must be addressed. Apart from the clinical symptoms they cause and the likelihood of successful treatment, it is known that infections and inflammation of the reproductive tract can affect spermatogenesis and, consequently, sperm parameters [3].

Firstly, substantial differences have emerged in the microbiota of patients who are positive or negative for STDs. The latter were characterized by a greater abundance of less fastidious, anaerobic and uncultivated bacteria [132].

There is growing evidence that *Ureaplasma* can have a negative impact on reproductive health. This view is supported by several studies and meta-analyses. A comprehensive meta-analysis by Farahani et al. [25] reported a significantly increased prevalence of *Ureaplasma*, mainly *U. urealyticum*, in infertile men compared to fertile controls, providing robust evidence for its pathogenic role. Several individual studies have corroborated this association across diverse populations. Yang et al. [32] found *Ureaplasma* significantly enriched in the semen of men with asthenozoospermia, while Okwelogu et al. [34] reported a notable relative abundance of Ureaplasma in azoospermic men. Similarly, Campbell et al. [41] observed an eight-fold higher abundance of *Ureaplasma* in men with NOA compared to fertile controls, although this difference did not reach statistical significance, possibly due to the small sample size.

Ureaplasma species, particularly *U. urealyticum*, are known to adhere to spermatozoa, impairing motility and membrane integrity, and inducing spermatozoa DNA fragmentation through oxidative stress [28,49]. The inflammatory response triggered by *Ureaplasma* colonization may further compromise the seminal microenvironment, contributing to reduced fertilizing capacity (Table 1).

Okwelogu et al. [34] also described how azoospermia was associated with a higher relative abundance of *Mycoplasma* genera. These can adhere so strongly to the surface of spermatozoa that they are not affected by washing procedures carried out prior to ART (Table 1).

**Figure 2:**
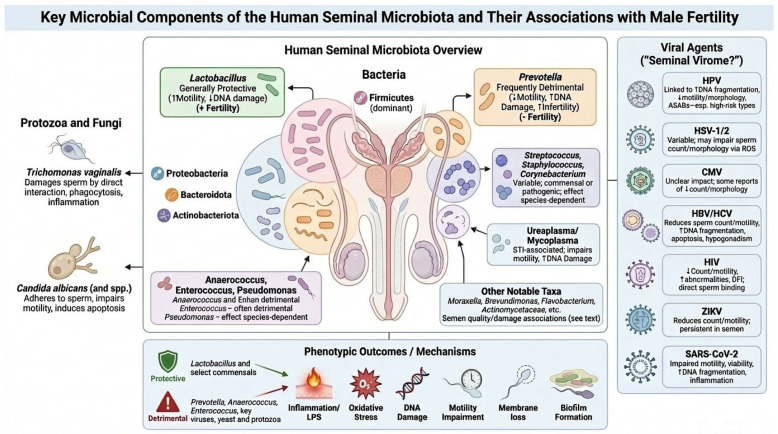
Graphical description of the key microbial components of the human seminal microbiota, alongside the main viruses, protozoa and fungi researched in this field and their associations with male infertility.

It is known how chronic *Chlamydia trachomatis* infection can lead to epididymitis, urethritis, and impaired spermatogenesis, with consequent reductions in spermatozoa count, motility, and increased DNA fragmentation [50,51]. However, NGS studies frequently fail to identify it as significantly associated with abnormal semen and male infertilty [25,30,40]. This underrepresentation may be associated with the obligate intracellular lifestyle of *Chlamydia*. Indeed, standard untargeted NGS workflows have produced unsatisfactory results, with fewer than 0.01% of sequenced reads being assigned to *Chlamydia*. (Supplementary Table S1, Figure 2, Table 1) [133].

Overall, the literature is characterized by substantial methodological heterogeneity, including differences in study populations, sequencing strategies, bioinformatic pipelines, and procedures for controlling contamination. These factors likely contribute to the inconsistent findings reported for several taxa, including *Lactobacillus*, *Staphylococcus*, and *Pseudomonas*. Future studies employing standardized methodologies, larger cohorts, longitudinal designs, and species-level characterization are essential in order to distinguish true biological associations from methodological variability.

## 5. Beyond Bacteria: Viral Agents Detected in Semen

The field of microbiota research is undoubtedly dominated by studies and analyses focusing on bacterial populations. However, it is now widely recognized that the microbiota is a much more complex entity, comprising not only bacteria but also viruses and eukaryotes (e.g., fungi and protozoa), that are likely to communicate with bacteria in complex ways. Nevertheless, evaluation of the human virome is rarely undertaken. Unlike bacteria and their 16S rRNA gene, viruses do not have shared sequences, which complicates and increases the cost of the NGS procedure. Therefore, most studies searching for a viral cause of male infertility focus on the effects of individual viral agents. An important conceptual distinction should be made when discussing the seminal virome. Unlike bacteria, for which resident microbial communities have been described within the male reproductive tract, the identification of stable viral communities remains largely unexplored. Consequently, the viruses discussed in this section are not necessarily permanent constituents of the seminal virome. Some viruses, such as HPV, HSV, and CMV, have been repeatedly detected within the male genital tract and may establish local persistence. Others, including HBV, HCV, HIV, ZIKV, and SARS-CoV-2, are more appropriately viewed as systemic pathogens that may reach semen through viral dissemination, local inflammation, or transient shedding. Nevertheless, their detection in seminal fluid and their potential effects on spermatozoa function and reproductive outcomes justify their consideration within the broader context of male reproductive microbiology.

This section will focus on viruses that have been more frequently identified as potentially causing seminal impairment. Other viruses that have been cited as possible causes of orchitis and impaired testicular function, such as *Varicella zoster*, *Mumps*, *Influenza*, *Coxsackie*, *Dengue*, *Rubella* and *Echovirus*, will be excluded (Figure 2).

### 5.1. Human Papillomavirus (HPV)

Human papillomavirus (HPV) is arguably the most extensively researched virus in the field of male infertility. It is the world’s most common sexually transmitted pathogen, notoriously correlated with cervical cancer and other malignancies of the genital and head and neck areas [134,135,136,137]. Its sexual transmission has always provided strong evidence for its presence within the male genital tract, where it has specifically been detected in the epididymis, vas deferens, seminal vesicles and prostate [21,138]. Therefore, it has long been hypothesized that HPV influences reproductive potential by impairing spermatozoa parameters. However, despite these efforts, no consensus has been reached [52].

The most frequently compromised seminal parameters in the presence of HPV positivity are spermatozoa motility, morphology and concentration, as well as abnormal seminal viscosity and pH [53,54,55,56,57,58,59,60,61]. Alongside these alterations, HPV infection appears significantly correlated with damage to spermatozoa DNA integrity. Studies by Moreno-Sepulveda et al. [62], Sun et al. [61] and Kato et al. [63] have found DFI rates higher than 30 to be associated with HPV positivity. Kato et al. specifically detected an increase in seminal superoxide dismutase (SOD) levels, suggesting the involvement of ROS in spermatozoa DNA impairment.

Moreover, several studies have highlighted the involvement of HPV infection in the production of ASABs. Garolla et al. [64] found a higher rate of ASABs in HPV-positive infertile men than in HPV-negatives. Subsequently, Foresta et al. [65] described an association between idiopathic asthenozoospermia, HPV infection, and the presence of ASABs.

These findings were consistent with those of Piroozmand et al. [66], who found that 17.4% of infertile men producing ASABs were also HPV-positive.

However, these results cannot be considered exhaustive or conclusive, as other authors have found no correlation between HPV positivity and spermatozoa impairment [67,68,69,70].

The lack of a consistent methodological approach is a key factor contributing to the observed heterogeneity in the literature, as well as to the variable size of the analyzed cohorts.

Alternatively, this may be because studies frequently failed to consider a fundamental issue: the difference between high-risk (hrHPV) and low-risk HPV (lrHPV). Indeed, it has been hypothesized that, as with their different involvement in carcinogenesis, hrHPV and lrHPV genotypes could affect reproductive potential differently. Despite the small number of studies focusing on this aspect, interesting results have emerged regarding the specific effects of hrHPV genotypes. Indeed, high levels of spermatozoa DNA fragmentation have been strongly associated with hrHPV in studies by Boeri et al. [71], Capra et al. [55] and Notari et al. [54]. A correlation with increased ROS production and spermatozoa impairment, including morphological alteration, during an hrHPV infection has also been described by Pérez-Soto et al. [72], who explained this effect by referring to the overexpression of the cytochrome P450 2E1 gene. Alterations in spermatozoa progressive motility [71] and spermatozoa count [66] have also been reported, with the latter correlation confirmed by a recent meta-analysis by Wang et al. [73].

However, no consensus has been reached, as other authors found no differences between hr and lrHPV [69].

Those who reject the idea that HPV plays a role in male infertility argue that its presence in semen is merely a consequence of desquamation of external genital keratinocytes [68].

The discovery of an association between HPV infection and alterations in the vaginal microbiota suggests the possibility of a similar interplay in males. However, research on this topic remains scarce and largely unsatisfactory. In 2021, Tuominen et al. [139] analyzed thirty-one semen samples and did not find any difference in alpha and beta diversity between HPV-positive and -negative samples. The only difference regarded the relative abundance of specific bacterial taxa: HPV-positives presented a higher relative abundance of families such as *Streptococcaceae*, *Peptostreptococcaceae*, *Veillonellaceae*, and *Moraxellaceae*. As well as the following genera: *Streptococcus*, *Serratia*, *Dialister*, and *Peptostreptococcus*. Notably, *Anaerococcus mediterraneensis* was also highly represented in the presence of HPV. On the contrary, in the absence of HPV, the phylum *Fusobacteria* was significantly more abundant.

It is necessary to highlight that semen parameters were not assessed in this study, and all the samples belonged to presumably fertile men (Table 1).

### 5.2. Herpes Simplex 1/2 (HSV1/2)

Another virus which has drawn attention due to its sexual transmission is Herpes simplex 1/2 (HSV1/2), with yet a few studies engaging on analyzing the effect of the virus in the onset of infertility.

To begin with, several studies have reported a higher prevalence of HSV infection in infertile men. However, there is no consensus on whether its detection is correlated with impairment of seminal parameters.

For instance, Salar et al. [74] reported a significantly higher rate of HSV1 and HSV2 detection in the seminal samples of infertile men than in those of fertile men. They also found that the presence of the virus was significantly correlated with lower spermatozoa count and a higher rate of abnormal morphology. Focusing on infertile individuals, spermatozoa count was significantly lower in the presence of HSV1/2.

A reduced spermatozoa count was registered also by Kotronias et al. [75], Komijani et al. [76], and Seyed et al. [77], who also found a correlation between viral detection and motility impairment, morphological abnormalities of head and neck, and higher apoptosis percentages, respectively.

Meanwhile, Kurscheidt et al. [78] found that a lower spermatozoa count was only present in the case of HSV1, whereas HSV2 was associated with hematospermia and a lower seminal volume. Bezold et al. [79] also described the latter, together with abnormal viscosity, as being strictly linked to prostatic and epididymal dysfunctions, and reported an impairment of spermatozoa concentration and motility.

Nonetheless, the meta-analysis by Guo et al. [22] did not show any significant difference in seminal parameters impairment between HSV-positive and negative individuals. Older studies, like that by Neofytou et al. [80], did not describe any impairment of spermatozoa parameters in HSV-positive samples.

The mechanisms by which HSV can damage spermatozoa are again linked with OS induction. In particular, it has been demonstrated how HSV1 infection can cause the dissociation of shelterin protein from spermatozoa telomere, which in turn cause their degradation through DNA repair machinery [81].

Moreover, the presence of HSV-DNA inside spermatozoa was demonstrated, in 1998 and then in 2008, by Kotronians et al. [75] and Bocharova et al. [82], respectively. Performing in situ hybridization, the two research groups detected both HSV1-DNA [75] and HSV2-DNA [75,82] in spermatozoa of infertile patients, opening to the alarming possibility of a viral effect on fetal genome (Table 1).

### 5.3. Cytomegalovirus (CMV)

HSV is not the only herpesvirus believed to play a role in the pathogenesis of infertility: Cytomegalovirus (CMV) is another example. Nevertheless, studies focusing on its association with male infertility do not unanimously suggest that the virus plays an active role.

CMV-DNA has been found in semen [79], and its presence was attested also in the prostate [21]. However, Salar et al. [74] found no association between viral DNA detection and spermatozoa aberrations. Cito et al. [83] reached the same conclusion, finding no association between CMV and male reproductive function. It should be noted that, in this case, CMV positivity was established only by serological analysis and not by identification of CMV DNA in semen samples.

Several other studies performed in the 1990s and at the beginning of the 21st century support the idea that CMV does not negatively impact semen quality or male fertility [79,80,84,85].

Fewer papers indicate an involvement of CMV infection in spermatozoa impairment. Analyzing seminal samples from a cohort of 150 infertile men, Namavar Jahromi et al. [86] found that morphology and spermatozoa count were statistically significantly altered in the CMV-positive samples. Furthermore, the viral copy number was higher in samples with impaired seminal parameters, with a study by Mohseni et al. [87] yielding comparable outcomes. This study involved screening for CMV DNA and conducting semen analyses on 200 men attending a fertility clinic for IVF procedures. CMV prevalence was once again found to be correlated with male infertility, resulting in a statistically significant reduction in sperm count [87].

As the other viruses described so far, it seems that CMV can bind to spermatozoa, along with infect immature spermatozoa, thereby exerting a gametotoxic effect [88] (Table 1).

### 5.4. Hepatitis B Virus (HBV)

The hepatitis B virus (HBV) is a major global public health concern, affecting an estimated 400 million people worldwide. Aside from its infamous role in acute and chronic hepatitis, liver cirrhosis and hepatocellular carcinoma, the presence of HBV in testis and semen has long been questioned in relation to male infertility and reproductive outcomes.

Indeed, the incidence of infertility has been shown to be higher in males with HBV, and several studies have demonstrated alterations to seminal parameters alongside the infection. Reductions in spermatozoa concentration, motility, and viability [89]; higher rates of morphological abnormalities, apoptosis, and DNA fragmentation [90]; loss of spermatozoa membrane integrity; altered cytokine expression [140]; and reduced fertilization capacity [90] have been observed and described in HBV-positive seminal samples. In particular, increased ROS concentration, reduced total antioxidant capacity, lipid peroxidation, caspase activation and phosphatidylserine externalization appear to be responsible for the aforementioned spermatozoa impairment following hepatitis B virus S (HBs) protein exposure [91]. HBs protein seems to be directly involved in the generation of oxidative stress. Indeed, Kang et al. [92] described how the exposure of spermatozoa to HBs was reflected in an augmentation of malondialdehyde, an indicator of OS, but also of apoptotic markers (caspases 3, 8, 9) leading to apoptosis, and a decrease in matrix metalloprotease, which damage mitochondrial integrity reducing spermatozoa motility.

The recent meta-analysis by Guo et al. [22] it is particularly useful in defining also the effect of HBV positivity on semen quality; they found a significant reduction in semen volume, concentration and viability, as well as a higher DFI.

Viral DNA has also been identified as integrated in spermatozoa genome causing genomic instability and chromosomal aberrations, raising the prospect of HBV-induced embryonal chromosomal defects [93] (Table 1).

### 5.5. Hepatitis C Virus (HCV)

Another hepatotropic virus, the hepatitis C virus (HCV), has also been suggested to play a role in male infertility. Some studies have pointed out the presence of HCV in semen samples and even its association with altered seminal parameters. In particular, chronic infected males analyzed by Hofny et al. [94] showed a reduction in spermatozoa count, spermatozoa motility and semen volume and a higher percentage of morphological abnormalities. The same significative impairments in chronic HCV patients were described by Safarinejad et al. [95], which also detected a higher percentage of spermatozoa chromosomal aberrations (i.e., disomy for chromosomes 18, X, and Y). Confirming these results was again the meta-analysis by Guo et al. [22], which described also an alteration in spermatozoa viability and an increase in DFI.

These aberrations are supposedly linked to the production of nitric oxide (NO) and ROS, the latter mainly induced by the viral proteins E1 and NS3 [96].

Moreover, HCV infection has been linked to a decrease serum level of luteinizing hormone (LH), follicle-stimulating hormone (FSH) and testosterone (T) levels, as well as a higher percentage of spermatozoa chromosomal aberrations [22] (Table 1).

### 5.6. Human Immunodeficiency Virus (HIV)

The presence of Human immunodeficiency virus (HIV) in semen is not questionable, as it functions as the principal factor of transmission during unprotected sexual intercourse.

Once again, in situ hybridization and immunohistochemistry allowed the identification of HIV-1 DNA inside spermatozoa, as well as the detection of viral particles directly attached to their surfaces [97,98]. Probably involved in this binding are CCR5 and CCR3, located respectively in the peri-acrosomal and post-acrosomal region of the spermatozoa head [99].

Hence, the presence of HIV in semen can covey as spermatozoa-associated viral particles, free virions, or infected leukocytes.

HIV infection can have a negative impact on male fertility both directly and indirectly. Indeed, the virus can replicate in the male genital tract, leading to increasing cytokine production, lymphocytes infiltration and interstitial fibrosis. A commonly diagnosed consequences in HIV patients is orchitis, which leads to ROS and DNA damage. Since these effects are more common in AIDS patients, the impact of HIV on spermatozoa parameters is presumably linked to the disease state.

Direct effects on spermatozoa have been described as a decrease in spermatozoa count, semen volume, and motility, and an increase in pH, DFI, and rates of abnormal morphology [22].

Reverse transcriptase inhibitors used in antiretroviral therapy have been associated with mitochondrial toxicity, which may impair ATP production and contribute to reduced spermatozoa motility [100] (Table 1).

### 5.7. ZIKA Virus (ZIKV)

The alleged sexually transmission of ZIKA virus (ZIKV) has brought up the hypothesis that its presence in semen could actively compromise spermatozoa parameters and male fertility.

Mead et al. found ZIKV RNA in 33% of the 1327 seminal samples analyzed and discovered that viral shedding could persist for more than three months. Other authors have hypothesized a much longer shedding period, that can last for up to 370 days in semen [101]. Later, the same authors highlighted how a longer shedding period was associated with a high seminal concentration of leukocytes and inflammatory cytokines. Again, the main consequences seem to be inflammation-dependent ROS production and OS.

ZIKV RNA was specifically found inside seminal leukocytes, epithelial cells, and spermatozoa [21]. The entry of ZIKV in spermatozoa has been described as probably involving Sertoli cells, which exhibit on their surfaces the viral receptor Axl, absent in spermatozoa. Its replication in Sertoli cells, Leydig cells, testicular macrophages, peritubular cells, but also immature and late germ cells or spermatozoa, could be the reason behind ZIKV-induced semen alterations [21].

With regard to seminal parameters, Joguet et al. [102] registered a decline in total spermatozoa count after Zika infection. Experiments performed on mice revealed abnormal testicular and epididymal function, which impaired spermatozoa count and motility [103] (Table 1).

### 5.8. Severe Acute Respiratory Syndrome Coronavirus 2 (SARS-CoV-2)

As strange as it may sound, the involvement of severe acute respiratory syndrome coronavirus 2 (SARS-CoV-2) in semen aberrations and fertility issues has been questioned and quite extensively investigated.

This hypothesis arises from the modality of viral entrance in host cells, in which cell receptor angiotensin-converting enzyme 2 (ACE2) and type II transmembrane serine protease (TMPRSS2), play a crucial role. These two membrane structures are highly expressed in testis, where severe sequelae like testicular atrophy, orchitis, inflammatory cell infiltration, germ cell apoptosis, and microthrombosis were correlated to SARS-CoV-2 infection.

ACE2 is mainly located in spermatogonia, seminiferous duct cells, Sertoli cells and Leydig cell, and since its expression is the highest in thirty years old males, the effect of SARS-CoV-2 infection on fertility raise quite a concern [104].

As no viral genome has been detected in testicular tissue through in situ hybridization; it is assumed that the damage described is an indirect consequence of the infection, strictly linked to inflammatory and immunological response.

Results concerning the presence of SARS-CoV-2 in seminal samples are not entirely coherent: while some studies have registered seminal viral shedding in patients suffering COVID-19 [105,106,107,108], others have not [109,110].

Interesting results emerged also from post-mortem investigations, even though performed in very small cohort. Yang et al. [111] and Achua et al. [112] analyzed testicular tissue samples belonging to patients who died from COVID-19. Even though SARS-CoV-2 was absent in the majority of samples, anomalies attributable to orchitis (i.e., seminiferous tubular injury, reduced number of Leydig cells, swelling, vacuolization, cytoplasmic rarefaction, detachment of the tubular basement membranes of Sertoli cells, and mild lymphocytic inflammation) were registered [111].

In any case, the meta-analysis by Guo et al. [22] concluded that SARS-CoV-2 infection had a detrimental effect on spermatozoa motility, viability and morphology, and resulted in an increase in spermatozoa DNA fragmentation.

Holtmann et al. registered a lower spermatozoa concentration, progressive motility, and count in infected patients in respect to healthy control [113].

The induction of OS through persistent inflammation, which is strongly associated with SARS-CoV-2, has been identified as the mechanism by which spermatozoa impairment is induced [81].

Interesting findings can be taken from the rare longitudinal studies existing on the subject: an infection longer than 30 days resulted in a significant reduction in spermatozoa total motility and raising of abnormal morphology spermatozoa [114] (Table 1).

## 6. What About Protozoa and Fungi?

Research on protozoa and fungi inhabiting the human body is exceedingly scarce, with these organisms being frequently excluded when the subject is microbiota.

About male infertility, available evidence remains limited and is mainly restricted to microorganisms already recognized as causes of other urogenital conditions, such as *Trichomonas vaginalis*, a common sexually transmitted pathogen, and *Candida albicans*, a frequent cause of genital mycosis. Even in this context, however, the available evidence is limited to a few case reports and small clinical and in vitro studies that do not permit any definitive conclusions to be drawn.

*Trichomonas vaginalis*, once considered irrelevant in male patients, was later found in the testes, urethra, epididymis and prostate, where it was found to cause urethritis, prostatitis and epididymo-orchitis [115]. Although only a few studies have been conducted on its effect on seminal parameters, the results so far suggest that the parasite could impairs spermatozoa function and quality, and that this effect could be linked to mechanical damage to the lining membrane of reproductive organs. Moreover, as described for other microorganisms till now, the *T. vaginalis* dependent impairment of reproductive function could also be linked to the activation of an inflammatory response.

It has also been described how *T. vaginalis* is able to phagocytize and agglutinate spermatozoa [116], and how proteins secreted by the protozoan could induce immobilization and death of spermatozoa. Electronic microscopy allowed the visualization of protozoan–spermatozoon interaction, which translated in agglutination, protrusions of cell membrane, and formation of channels between parasite membrane and spermatozoa plasma [117]. The glycoprotein-mediated interaction between protozoan and spermatozoa could lead to alterations in their horizontal movement and obstruction of spermatozoa–egg binding. Another consequence may be the retrieval of neutrophiles which can phagocyte spermatozoa [118].

The recent meta-analysis by Zhang et al. revealed that infertile men and women showed a higher incidence of Trichomoniasis in respect to fertile patients [118].

Hosseini et al., screening 181 male with infertility issues plus 16 controls, found only one case of Trichomoniasis, which was associated with 0.3% normal spermatozoa with 19% motility, number that could not allow the achievement of a satisfactory conclusion [119].

Long before, Gopalkrishnan et al. described a significant alteration of spermatozoa motility, viability, morphology and seminal viscosity associated with the infection [120].

Quite a different approach was carried out in the study by El Saftawy et al., which consisted in incubating for 24–48 h seminal samples with *Trichomonas vaginalis.* This resulted in a reduction in seminal pH and spermatozoa motility, as well as an augmentation of seminal protein, which has been linked to an increase in seminal viscosity. The increase in DFI registered in this study was however non-significant [121].

Details about the mechanism involved in spermatozoa impairment emerged from the study by Zhang et al., who analyzed the effect of the exposure of the excretory secretory proteins of *T. vaginalis* (TvESPs) on spermatozoa [122]. Authors registered several abnormalities, including a dose and time-dependent reduction in spermatozoa motility, as well as an increase in spermatozoa apoptosis and mortality, also dependent of dose and time. A disruption of acrosome structure was also registered. These findings were accompanied by in vivo experiments on mice which demonstrated a negative impact of TvESPs on spermatozoa fertilization ability and development of fertilized egg, as well as a downregulation of apoptosis-related factor.

Some interesting evidence has emerged from a few case reports. In one study, a 34-year-old man was found to have NOA, which was probably caused by epididymal trichomoniasis, resulting in an arrest of spermatozoa cell maturation [123]. Similarly, Gong et al. detected the presence of *T. vaginalis* in the testes of a patient with NOA and hypothesized that the protozoan could have played a role in inducing the condition [124].

*Candida* spp. have only recently been associated with impaired male reproductive parameters.

*Candida albicans* is the most common fungal urogenital pathogen and has been reported in association with impaired spermatozoa quality in some experimental and clinical studies. The first evidence of this came from in vitro studies, like that from Burrello et al. [125], who incubated 13 normozoospermic samples with increasing concentrations of *C. albicans*. The results showed concentration-dependent impairment of spermatozoa motility and mitochondrial membrane potential, as well as an increased phosphatidylserine externalization, indicating apoptosis. No statistically significant association was found regarding DNA fragmentation, although fragmentation was nonetheless observed. These findings were corroborated by Sasikumar et al. [126], which, applying the same approach showed how the presence of *C. albicans* and *C. tropicalis* was associated with a decline in motile sperms.

Later, Castrillón-Duque et al. [127] described a time and concentration-dependent alteration in viable and motile spermatozoa following semen incubation with *C. albicans* and *C. glabrata*. Despite being statistically non-significant, an increase in late apoptosis and DNA fragmentation was induced by *C. albicans*, while *C. glabrata* boosted early apoptosis.

Furthermore, exposure to mannose reduced motility impairment, which, alongside the presence of mannose in yeasts and mannose receptors on spermatozoa, may explain the observed interaction and agglutination.

These findings were partly supported by electron microscopy analyses, which have shown the presence of *Candida albicans* attached to the spermatozoa surface and have described associated ultrastructural changes [128] (Figure 2, Table 1).

## 7. Anatomical Origins of the Seminal Microbiota

One issue that warrants further investigation is the origin of the microorganisms found in semen, as this is closely linked to the anatomy of the male reproductive tract. Semen is, in fact, a complex fluid, which, after the production of spermatozoa in the seminiferous tubules of the testicles, passes through different anatomical sites: first into the rete testis, then the efferent ducts, epididymis, and vas deferens, where it mixes with secretions from the seminal vesicles, prostate gland, and bulbourethral glands, ultimately forming semen that is ejaculated through the urethra [3].

Therefore, it has been hypothesized that all anatomical sites of the male reproductive tract, extending from the upper genital tract to the distal urethra, may serve as original niches for the microorganisms detected in semen. A separate consideration is the presence of microorganisms originating from other body sites, introduced through sexual practices (e.g., from the vagina or oral cavity) or potentially derived from the gastrointestinal tract via systemic dissemination or microbial translocation.

Depending on their location and origin, microorganisms may exert different effects on spermatozoa. Those residing in the urethra or external genital tract are likely to have a lesser impact on spermatozoa than those inhabiting the upper genital tract, primarily because of differences in the duration of exposure.

To begin with, the testicular microenvironment, formerly thought to be free of microbial presence, now appears to be susceptible to microbial colonization. Indeed, Suarez Arbelaez et al. [141] described a vasectomy-dependent change in seminal microbiota, which could indirectly suggest the presence of a testicular/epididymal-specific community. The authors described a reduction in alpha diversity following a vasectomy procedure, with a specific decrease in *Sphingomonas*, *Brevundimonas*, and *Paracoccus*, and a significant increase in *Actinobacteria* and *Corynebacterium*. The “absence” of spermatozoa has been described as associated with a peculiar microbiota also by Alfano et al. [142], who analyzed testicular samples belonging to a group of patients affected by NOA. The comparison with healthy testicular tissue samples revealed an increased bacterial load in azoospermic individuals, alongside a decrease in *Proteobacteria* and *Bacteroidetes*, compared to normal samples, in which *Actinobacteria* and *Firmicutes* were also among the most prevalent phyla [142].

Subsequent NGS testing of testicular spermatozoa from infertile men revealed ten testis-specific bacterial genera: *Prevotella*, *Blautia*, *Clostridium*, *Cellulosibacter*, *Collinsella*, *Robinsoniella*, *Prolixibacter* and *Wandonia* [143].

Regarding the epididymis, where spermatozoa gain motility and fertilizing capacity, the literature is even scarcer, with no studies specifically focused on examining its microbiota composition.

In contrast, several studies have investigated prostate microbiota; however, these have predominantly focused on its association with prostatic diseases, including prostate cancer, prostatitis, and benign prostatic hyperplasia.

Nevertheless, some interesting conclusions can still be drawn from these investigations. In particular, Okada et al. [144] found a substantially different microbiota in urine samples compared to prostate samples belonging to patients affected by benign prostatic hyperplasia, which seems to indicate the existence of a prostate-specific community. Specifically, the microbiota of the prostate was characterized by a higher beta diversity than that found in the urine, and *Proteobacteria*, *Actinobacteria*, *Bacteroides* and *Firmicutes* were the dominant phyla in both sample type. Moreover, the authors revealed through in situ hybridization (ISH) that bacteria were localized in the prostatic duct, suggesting a urinary tract origin rather than via bloodstream.

These findings were corroborated by those of Cavaretta et al. [145], which analyzed paraffin-embedded tissue samples from radical prostatectomy. Again, the most abundant phyla were *Actinobacteria*, *Firmicutes*, and *Proteobacteria*, with genera including *Corynebacterium*, *Propionibacterium*, and *Staphylococcus*.

With regard to the viral counterpart, HPV-DNA has been identified in prostatic tissue, where it has been mainly investigated in relation to prostate carcinogenesis rather than male infertility [146].

Due to the difficulty and invasiveness of sampling procedures, the microbiota of the seminal vesicles and bulbourethral glands has been investigated only rarely, and almost exclusively in the context of pathological conditions. The only authors examining this niche were Lei et al. [147], identifying *Firmicutes*, *Bacteroidetes*, *Proteobacteria*, *Actinobacteria*, and *Fusobacteria* as the most abundant phyla, and *Bacteroides*, *Lactobacillus*, *Bifidobacterium*, and *Faecalibacterium* as the most represented genera. However, regarding genera, contamination with urine cannot be excluded. Among the viral agents, HSV2 DNA has been found in seminal vesicles [21].

The bacteria found in the urethra may not necessarily be residents of this anatomical region but may originate from the bladder or other urogenital niches, since voided and catheterized urine share dominant genera and some studies suggest interlinked urogenital communities [148,149]. Evidence from different studies identified *Lactobacillus*, *Streptococcus*, *Sneathia*, *Veillonella*, *Corynebacterium*, and *Prevotella* in male urethral or urinary microbiota [148,149,150].

Contamination from the penis’s skin microbiota is also plausible, because penile skin and coronal sulcus communities are often dominated by *Corynebacterium* and *Staphylococcus*, and circumcision substantially alters their relative abundance [149].

## 8. Microbiota Exchange and Sexual Partner Interactions

Sexual activity undoubtedly directly impacts the composition of genital microbiota, with factors such as frequency, contraceptive methods, and specific sexual practices (i.e., oral or anal sex) influencing not only the diversity and abundance of microbial communities but also the exchange of microorganisms between partners. The predictable outcome of this exchange, at least when we zoom in only on heterosexual intercourse, is reproductive health.

A change in seminal microbiota can be observed in conjunction with the sexual debut, after which an increase in the diversity and concentration of bacterial species has been observed [132]. In general, semen is less rich but more diverse in bacterial species than the vagina, with about 85% of shared taxa, including species as *Gardnerella vaginalis*, *Lactobacillus iners*, *Lactobacillus japonicus*, *Lactobacillus jensenii*, and *Lactobacillus agilis* [34,151]. However, as recently described by Okwelogu et al. [34], the sharing of genital microbial communities in stable couples is not obvious. The authors analyzed the seminal and vaginal microbiota of twenty-four couples who experienced an unsuccessful IVF attempt and twelve couples with a successful IVF outcome.

No strict correspondence was observed between the bacterial taxa present in partners within each couple, particularly with regard to their relative abundance. This lack of concordance may be explained by sexual practices, such as oral or anal intercourse preceding vaginal sex, as well as by environmental and lifestyle factors, including smoking, personal hygiene, and alcohol consumption, which may affect individuals differently. Unfortunately, research on this topic remains remarkably limited, and current knowledge is based on only a few studies.

## 9. Conclusions and Future Directions

Research on the seminal microbiota is uncovering an increasingly complex picture, with important knowledge gaps remaining. Studies conducted to date have identified several viral agents and bacterial taxa that appear to be involved in male fertility; however, their precise roles are often poorly defined, and many questions remain unanswered.

Designing longitudinal studies capable of capturing physiological and pathological changes and elucidating the causal relationships between infertility and the microbiota will be of paramount importance. Such studies may also clarify whether lifestyle modifications or specific therapeutic interventions can improve or impair reproductive function.

Ideally, once the role of each component of the bacterial and viral microbiota in impairing male fertility is clearer, it may become possible to develop advanced diagnostic tools, such as NGS panels, capable of accurately identifying men at risk of infertility and providing valuable insights for couples facing the prolonged and psychologically challenging condition of idiopathic infertility.

A better understanding of the bacterial communities inhabiting healthy and dysfunctional male urogenital tracts, together with the precise roles of viruses, protozoa, and fungi, could also pave the way for novel therapeutic strategies. In the future, microbiota-targeted interventions, including prebiotics and probiotics, may be employed to restore microbial homeostasis and improve reproductive health. However, evidence supporting their efficacy as a stand-alone therapeutic approach remains limited [26]. Such approaches could complement the antibiotic therapies already recommended by the European Association of Urology (EAU), which have been shown to improve seminal parameters in the treatment of specific genital tract infections (e.g., *Chlamydia trachomatis* and *Ureaplasma urealyticum*) [152].

At present, however, the available evidence is insufficient to support routine clinical screening for most bacterial and non-bacterial microorganisms, and larger prospective studies are required to establish their diagnostic, prognostic, and therapeutic significance.

## Figures and Tables

**Table 1:** Summary table of the main associations between microorganisms (bacteria, viruses, protozoa, and fungi) and male fertility, highlighting reported alterations in semen parameters, proposed pathogenic mechanisms, and the overall consistency of evidence available in the scientific literature.

Microorganisms	Main Reported Association with Fertility	Semen Abnormalities Reported	Proposed Mechanisms	Evidence Consistency	Key References
*Prevotella* spp.	Frequently associated with infertility and dysbiosis	Reduced motility, oligospermia, abnormal morphology, increased bacterial load	LPS-induced inflammation, oxidative stress, competition with Lactobacillus	High consistency across studies	[7,28,31,32,34,35,39,40]
*Lactobacillus* spp.	Generally associated with normozoospermia and better fertility outcomes	Improved motility, lower DNA fragmentation; conflicting findings in asthenozoospermia	Production of lactic acid and SCFAs, pathogen exclusion, ROS regulation	Moderate consistency; species-dependent effects	[28,31,32,34,36,40,42,43,44,45,46,47]
*Pseudomonas* spp.	Variable association	Altered spermatozoa count in some studies	Possible toxin production and inflammatory effects	Low consistency	[7,28,29,30,33]
*Staphylococcus* spp.	Both protective and detrimental associations reported	Asthenozoospermia, hyperviscosity, altered motility	Biofilm formation, inflammatory activation; possible contamination during collection	Contradictory findings	[7,31,32,33,34,35,39,46]
*Anaerococcus* spp.	Often associated with poor semen quality	Reduced concentration, motility, and semen volume	Dysbiosis-associated inflammation	Moderate consistency	[3,7,30,31,32,48]
*Enterococcus* spp.	Frequently enriched in infertile men	Azoospermia, high DNA fragmentation, altered spermatozoa quality	Biofilm production, inflammatory damage	Moderate consistency	[30,33,34,36,39]
*Ureaplasma urealyticum*	Strongly associated with infertility	Reduced motility, membrane damage, increased DNA fragmentation	Adhesion to spermatozoa, oxidative stress, inflammation	High consistency including meta-analysis	[25,28,32,34,41,49]
*Mycoplasma* spp.	Associated with azoospermia and spermatozoa dysfunction	Reduced spermatozoa quality and ART impairment	Strong adhesion to spermatozoa cells, persistent colonization	Moderate consistency	[34]
*Chlamydia* *trachomatis*	Known STI linked to reproductive tract inflammation	Reduced count, motility, increased DNA fragmentation	Chronic inflammation, epididymal damage	Moderate clinical evidence but inconsistent NGS detection	[25,30,40,50,51]
Human papillomavirus (HPV)	Most studied seminal virus; association remains debated	Reduced motility, abnormal morphology, reduced concentration, increased DFI, ASAB positivity	Oxidative stress, immune activation, spermatozoa DNA damage	Contradictory overall, stronger evidence for hrHPV	[52,53,54,55,56,57,58,59,60,61,62,63,64,65,66,67,68,69,70,71,72,73]
Herpes simplex virus (HSV-1/2)	Frequently detected in infertile men	Reduced spermatozoa count, motility impairment, abnormal morphology	Oxidative stress, telomere damage, direct spermatozoa infection	Moderate inconsistency	[74,75,76,77,78,79,80,81,82]
Cytomegalovirus (CMV)	Uncertain role in infertility	Reduced spermatozoa count and morphology in some studies	Gametotoxic effects, direct interaction with spermatozoa cells	Low consistency	[74,79,80,83,84,85,86,87,88]
Hepatitis B virus (HBV)	Strong association with impaired semen quality	Reduced concentration, motility, viability; increased apoptosis and DFI	ROS production, mitochondrial dysfunction, DNA integration	Moderate consistency	[22,89,90,91,92,93]
Hepatitis C virus (HCV)	Associated with chronic spermatozoa impairment	Reduced count, motility, morphology, increased chromosomal abnormalities and DFI	ROS/NO production, endocrine disruption	Moderate consistency	[22,94,95,96]
Human immunodeficiency virus (HIV)	Associated with impaired reproductive function	Reduced count, motility, semen volume; increased DFI	Chronic inflammation, orchitis, ART-related mitochondrial toxicity	Moderate consistency	[22,97,98,99,100]
Zika virus (ZIKV)	Emerging evidence of reproductive tract involvement	Reduced spermatozoa count and motility	Testicular inflammation, Sertoli/Leydig cell infection	Limited evidence	[21,101,102,103]
SARS-CoV-2	Associated with transient semen impairment	Reduced motility, viability and morphology; increased DFI	Systemic inflammation, oxidative stress, orchitis-like damage	Moderate but heterogeneous evidence	[22,81,104,105,106,107,108,109,110,111,112,113,114]
*Trichomonas vaginalis*	Associated with infertility and spermatozoa dysfunction	Reduced motility, altered viscosity, apoptosis, spermatozoa agglutination	Inflammatory response, direct spermatozoa interaction, secreted proteins	Moderate consistency	[115,116,117,118,119,120,121,122,123,124]
*Candida* *albicans*	Associated with reduced spermatozoa quality in vitro	Reduced motility, mitochondrial dysfunction, apoptosis, agglutination	Adhesion to spermatozoa surface, induction of apoptosis	Limited mainly to in vitro evidence	[125,126,127,128]

## Data Availability

No new data were created or analyzed in this study. Data sharing is not applicable to this article.

## Supplementary Materials

The following supporting information can be downloaded at: https://www.mdpi.com/article/10.3390/biomedicines14071557/s1, Supplementary Table S1: Characteristics of studies performed on seminal samples analyzing bacterial microbiota using Next Generation Sequencing (NGS) methods, from 2013 to 2025.
