# Supplementary material for: Unfolding the Semen Microbiota: Implications for Male Infertility
Source: Biomedicines. 2026 Jul 11;14(7):1557. doi: 10.3390/biomedicines14071557 (PMC13407389; doi:10.3390/biomedicines14071557)
Supplement: Supplementary file 1 [file biomedicines-14-01557-s001.zip › biomedicines-4359367-supplementary.pdf]

<table>
<tr><th>Authors</th><th>Year</th><th>Method</th><th>Hypervariable regions</th><th>N° of patients</th><th>Cohort characteristics</th><th colspan="2">Most represented bacterial taxa</th></tr>
<tr><td rowspan="4">Hou et al. [3]</td><td rowspan="4">2013</td><td rowspan="4">Roche 454 GS-FLX (pyrosequencing)</td><td rowspan="4">V1-V2</td><td rowspan="4">77</td><td>Healthy subjects (n= 19)</td><td colspan="2" rowspan="4">PHYLA:
Firmicutes, Proteobacteira, Actinobacteria, Bacteroidetes

GENERA:
Lactobacillus, Corynebacterium, Streptococcus, Staphylococcus, Prevotella, Finegoldia, Anaerococcus, Peptoniphilus, Incertae Sedis XI (family), Veillonella, Pelomonas, Porphyromonas, Acidovorax, Atopobium, Ureaplasma, Bradyrhizobium, Aerococcus, Gemella, Granulicatella, Clostridiales, Cloacibacterium, Ralstonia.</td></tr>
<tr><td>Asthenozoospermia (n= 10)</td></tr>
<tr><td>Oligoasthenozoospermia (n= 23)</td></tr>
<tr><td>Oligoasthenozoospermia and azoospermia (n= 25)</td></tr>
<tr><td rowspan="2">Weng et al. [28]</td><td rowspan="2">2014</td><td rowspan="2">Illumina MiSeq</td><td rowspan="2">V4</td><td rowspan="2">96</td><td>Normal spermatozoa (n= 36)</td><td>GENERA:
Lactobacillus, Gardnerella, Propionibacterium, Atopobium

SPECIES:
Lactobacillus crispatus, Gardnerella vaginalis, Lactobacillus acidophilus, Uncultured gardnerella sp., Atopobium vaginae, Pseudomonas sp. ps10-13</td><td rowspan="2">GENERA:
Lactobacillus, Pseudomonas, Prevotella, Gardnerella, Rhodanobacter, Streptococcus, Finegoldia, Haemophilus

SPECIES:
Lactobacillus iners, uncultured Prevotella sp., uncultured Gardnerella sp., Lactobacillus sp., uncultured Pseudomonas sp., Prevotella bivia</td></tr>
<tr><td>Abnormal sperm: volume (n= 10), concentration (n= 13), motility (n= 12), morphology (n= 44), ASAB (n= 10), leukocytospermia (n= 18)</td><td>GENERA:
Prevotella, Pseudomonas, Haemophilus, Aggregatibacter,
Finegoldia, Rhodanobacter, Corynebacterium, Streptococcus, Campylobacter, Peptoniphilus, Brevibacterium, Pasteurella, Sphingobium, Dermococcus, Aggregatibacter, Varibaculum

SPECIES:
Prevotella bivia, Haemophilus parainfluenzae, Arthrobacter sp. Zn12, Sphingobium estrogenivorans, Varibaculum cambirense, Prevotella sp. BV3C7, Actinomyces sp., Prophyromonas somerae.</td></tr>
<tr><td>Chen et al. [29]</td><td>2018</td><td>Illumina HiSeq 2000 platform</td><td>V4</td><td>17</td><td>Healthy subjects (n= 5)</td><td>GENERA:
Alicyclobacillus, Amaricoccus, Anaeromyxobacter, Aquicella, Arsenicicoccus, Azospirillum, Chitinimonas, Chlorobacillus, Coprococcus, Desulfovibrio, Dokdonella, Gallionella, Geobacter, Helicobacter, Idiomarina, Kaistia, Kribbella</td><td>PHYLA:
Firmicutes, Proteobacteria, Bacteroidetes, Actinobacteria</td></tr>
</table>

					Obstructive azoospermia (n= 6)	*GENERA:* *Solibacillus, Campylobacter, Campylobacteraceae, Plesiomonas, Alicyclobacillus*	*GENERA:* *Alicyclobacillus, Amaricoccus, Anaeromyxobacter, Aquicella, Arsenicicoccus, Azospirillum, Chitinimonas, Chlorobaculum, Coprococcus, Desulfovibrio, Dokdonella, Gallionella, Geobacter, Helicobacter, Idiomarina, Kaistia, Kribbella*	*GENERA:* *Lactobacillus, Prevotella, Proteus, Pseudomonas, Veillonella, Corynebacterium, Rhodococcus, Staphylococcus, Bacillus*
					Non-obstructive azoospermia (n= 6)	*GENERA:* *Sneathia, Lysobacter sp*		
**Monteiro et al. [30]**	2018	Ion Torrent (Ion PGM)	V3-V6	118	Healthy subjects (n=29)	*GENERA:* *Enterococcus, Lactobacillus, Staphylococcus, Anaerococcus, Peptoniphilus*	*PHYLA:* *Firmicutes, Proteobacteria, Actinobacteria, Bacteriodetes, OD1* *FAMILIES:* *Caulobacteraceae, Pasteurellaceae, Enterobacteriaceae, Flavobacteriaceae, Actinomycetaceae* *GENERA:* *Enterococcus, Staphylococcus, Anaerococcus, Corynebacterium, Peptoniphilus, Propionibacterium, Aerococcus, Facklamia, Pseudomonas, Klebsiella, Haemophilus, Aggregatibacter*	
					Oligoasthenoteratozoospermia (n= 45)	*GENERA:* *Enterococcus, Pseudomonas, Klebsiella, Aerococcus, Flackamia*		
					Asthenoteratozoospermia (n= 15)	*FAMILIES:* *Gemellaceae* *GENERA:* *Enterococcus, Staphylococcus, Anaerococcus, Pseudomonas, Klebsiella*		
					Semen hyperviscosity (n= 29)	*PHYLA:* *Proteobacteria* *GENERA:* *Pseudomonas, Klebsiella, Enterococcus, Acinetobacter, Neisseria*		
**Baud et al. [31]**	2019	Illumina MiSeq	V1-V2	94	Normal spermatozoa (n= 24)	*GENERA:* *Staphylococcus, Lactobacillus*	*PHYLA:* *Actinobacteria, Bacteroidetes, Firmicutes, Proteobacteria*	

					Abnormal spermatozoa (n= 68):<br>(count, concentration, progressive motility, total motility, morphology)	*GENERA:*<br>*Prevotella*	*GENERA:*<br>*Corynebacterium, Prevotella, Lactobacillus, Streptococcus, Staphylococcus, Planococcusaceae, Finegoldia, Haemophilus, Burkholderia*	
**Yang et al. [32]**	2020	Illumina HiSeq<br>2500 system	V1–V2	159	Healthy controls (n= 58)	*GENERA:*<br>*Pelomonas, Propionibacterium, Bosea, genosp, Bosea, Afipia, Sphingomonas, Vogesella, Brevibacillus, Xylanimicrobium, Flexispira, Pedomicrobium, Phyllobacterium, Aquimonas, Dietzia, Sediminibacterium, Mycobacterium, Eikenella.*<br><br>*SPECIES:*<br>*Brevibacterium aureum, Propionibacterium acnes, Corynebacterium simulans, Eubacterium dolichum, and Bacillus thermoamylovorans*		*PHYLA:*<br>*Proteobacteria, Firmicutes, Actinobacteria, Bacteroidetes, Fusobacteria*<br><br>*GENERA:*<br>*Lactobacillus, Corynebacterium, Acinetobacter, Prevotella, Enterococcus, Veillonella, Streptococcus, Porphyromonas, Sneathia, Pelomonas*
					Oligoasthenospermia (n= 22)	** GENERA:*<br>*Ralstonia, Oscillospira, Parabacteroides, Lachnospira, Phascolarctobacterium, Chryseobacterium, Zoogloea, Ruminococcus, Stenotrophomonas, Actinoplanes, Mycoplana, Delftia, Sneathia, Megasphaera, Atopobium, Faecalibacterium, Bacteroides, Lactobacillus.*<br><br>**SPECIES:*<br>*Bacteroides uniformis, Stenotrophomonas panacihumi, Bacteroides*	*GENERA*<br>*Lactobacillus, Bacteroides, Delftia, Sneathia, Enhydrobacter, Anaerococcus, Mycoplana, Finegoldia, Stenotrophomonas, Methylobacterium, Coprobacillus, Aerococcus, Atopobium, Chryseobacterium, Kocuria, Megasphaera, Ralstonia, Achromobacter, Erwinia, Ureaplasma, FilifactoR*<br><br>*SPECIES:*	

						plebeius, Prevotella copri, and Faecalibacterium prausnitzii	*Prevotella copri, Saccharopolyspora hirsuta, Kocuria palustris, Prevotella nigrescens, Porphyromonas endodontalis, Lactobacillus coleohominis, Bacteroides barnesiae, Lactobacillus iners*	
					Asthenospermia (n= 58)	**GENERA: Sneathia, Ralstonia, Ureaplasma, Bacteroides, Chryseobacterium, Aerococcus, Enhydrobacter, Methylobacterium, Anaerococcus, Stenotrophomonas, Mycoplana, Delftia, Finegoldia, Corynebacterium, Lactobacillus (at the genus level),*<br><br>**SPECIES Saccharopolyspora hirsute, Acinetobacter lwoffii, Lactobacillus iners*		
					Azoospermia (n= 8)	*No significant differences compared to healthy controls*		
					Oligospermia (n= 13)	*No significant differences compared to healthy controls*		
**Okwelogu et al. [34]**	2021	Illumina NextSeq 500	V4	36	Positive IVF	*SPECIES: Lactobacillus jensenii, Faecalibacterium*		
					Negative IVF	*PHYLA: Proteobacteria*<br><br>*GENERA Prevotella, Escherichia, Bacteroides,*		

**Garcia-Segura et al. [36]**	2022	Illumina MiSeq	V1-V9	56	Normozoospermic semen donors (n= 14)	*FAMILIES:*<br>*Propionibacteriaceae*<br><br>*GENERA:*<br>*Cutibacterium, Rhodopseudomonas, Oligotropha*	*PHYLA:*<br>*Firmicutes, Proteobacteria, Actinobacteria, Bacteroidetes*<br><br>*GENERA:*<br>*Finegoldia, Peptoniphilus, Anaerococcus, Campylobacter, Streptococcus, Staphylococcus, Moraxella, Prevotella, Ezakiella, Corynebacterium, Lactobacillus*
					Idiopathic normozoospermic infertile patients (n= 42)	*FAMILIES:*<br>*Phylobacteriaceae, Vibrionaceae,*	
**Bukharin et al. [33]**	2022	Illumina MiSeq	V1-V2, V3-V4	72	Healthy subjects (n= 30)	*GENERA:*<br>*Staphylococcus, Corynebacterium, Enterococcus, Neisseria, Veillonella,*	
					Infertile subjects (n= 42)	*GENERA:*<br>*Staphylococcus, Corynebacterium, Enterococcus, Streptococcus, Escherichia, Actinomyces, Bacillus, Klebsiella, Pseudomonas, Propionibacterium*	
**Gachet et al. [7]**	2022	Illumina MiSeq	V1-V3	91	Normozoospermic infertile patients (n= 38)	*Mobiluncus, Finegoldia, Cutibacterium, Gordonia*	*GENERA:*<br>*Prevotella, Finegoldia, Pseudomonas, Peptinophilus, Streptococcus , Anaerococcus, Corynebacterium*
					Infertile patients with abnormal spermatozoa parameters (count, motility, morphology) (n=53)	*Haemophilus*	
**Cao et al. [39]**	2023	Illumina MiSeq	V3-V4	53	Healthy subjects (n= 12)		*PHYLA:*<br>*Firmicutes, Bacteroidetes, Actinobacteriota, unidentified phyla*<br><br>*GENERA:*<br>*Lactobacillus, Prevotella, Finegoldia, Staphylococcus, Streptococcus, Ureaplasma, other unidentified bacteria*
					Asthenzoospermia (n= 12)	*GENERA:*<br>*Staphylococcus*	
					Oligospermia (n= 6)	*GENERA:*<br>*Bacillus*	
					Severa oligospermia or azoospermia (n= 9)		
					Semen hyperviscosity (n= 14)	*GENERA:*<br>*Staphylococcus*	
**Campbell et al. [41]**	2023	Illumina MiSeq	V1-V2	33	Healthy subjects (n= 19)	*PHYLA:*<br>*Proteobacteria, Firmicutes*<br><br>*GENERA:*	*GENERA:*<br>*Enterococcus*

						Ideonella, Raoultella, Sneathia	
					Idiopathic NOA (n= 14)	*GENERA:*<br>*Escherichia/Shigella, Ureaplasma*	
**Chen et al. [37]**	2023	Illumina NovaSeq 6000	V3-V4	60	Helahty subjects (n= 30)	*GENERA:*<br>*Bifidobacterium, Ruegeria*	*PHYLA:*<br>*Bacteroidota, Firmicutes, Proteobacteria*<br><br>*GENERA:*<br>*Bacteroides, Prevotella, Muribaculaceae, Lactobacillus, Faecalibacterium, Escherichia-Shigella*
					NOA (n= 30)	*GENERA:*<br>*Bacteroides, Escherichia–Shigella, Ruegeria, Donghicola*	
**Fu et al. [38]**	2024	Illumina NovaSeq 6000	V3-V4 or V4-V5	28	Healthy subjects	*PHYLA:*<br>*Firmicutes, Bacteroidota, Proteobacteria, Actinobacteriota, Campylobacterota*<br><br>*GENERA:*<br>*Fenollaria, Campylobacter, Peptoniphilus, Negativicoccus, Prevotella, Porphyromonas, Muribaculaceae, Bacteroides, Mobiluncus, Varibaculum*<br>.	
**Osadchyi et al. [47]**	2024	Illumina MiSeq	V1-V2	73	Normozoospermic (n= 42)	*Staphylococcus hominis, Pseudomonas putida*	*SPECIES:*<br>*Enterococcus faecalis, Corynebacterium tuberculostearicum, Lactobacillus iners, Staphylococcus epidermidis, Finegoldia magna*
					Abnormal spermatozoa parameters (n= 31)	*Peptoniphilus coxii, Lactobacillus iners, Paraburkholderia phenazinium, Pseudomonas fluorescens, Pseudomonas stutzeri*	
**Mowla et al. [40]**	2025	MiSeq	V1-V2	223	Healthy controls (n= 63)	*GENERA:*<br>*Streptococcus, Prevotella, Lactobacillus/Gardnerella*	
					Male partners in couples with recurrent pregnancy loss (n= 46)		
					Men with male factor infertility (n= 58)		
					Male partners of couples with unexplained infertility (n= 56).		
**Yao et al. [35]**	2025	Illumina NextSeq 2000	5R (5 regions: V2, V3, V5, V6, V8)	40	Healthy subjects (n= 14)	*SPECIES:*<br>*Providencia vermicola, Bacillus methylotrophicus, Enterobacter cloacae, Enterobacter asburiae*	*PHYLA:*<br>*Proteobacteria, Firmicutes, Bacteroidetes, Actinobacteria*

					Idiopathic infertility (n= 26)	*SPECIES:*<br>*Providencia rettgeri, Prevotella bivia, Prevotella timonensis, Streptococcus mitis Providencia alcalifaciens, Acinetobacter schindleri Enterococcus*	

Supplementary Table 1. Characteristics of studies performed on seminal samples analyzing bacterial microbiota using Next Generation Sequencing (NGS) methods, from 2013 to 2025.

Supplementary Table 1. Characteristics of studies performed on seminal samples analyzing bacterial microbiota using Next Generation Sequencing (NGS) methods, from 2013 to 2025.
